# Effect of Precursor Purge Time on Plasma-Enhanced Atomic Layer Deposition-Prepared Ferroelectric Hf_0.5_Zr_0.5_O_2_ Phase and Performance

**DOI:** 10.1021/acsomega.5c01112

**Published:** 2025-05-14

**Authors:** Yong Kyu Choi, Kristina Holsgrove, Andrea Watson, Benjamin L. Aronson, Megan K. Lenox, Liron Shvilberg, Chuanzhen Zhou, Shelby S. Fields, Shihao Wang, Stephen J. McDonnell, Amit Kumar, Jon F. Ihlefeld

**Affiliations:** 1 Department of Materials Science and Engineering, 2358 University of Virginia, Charlottesville, Virginia 22904, United States; 2 School of Mathematics and Physics, 1596 Queen’s University Belfast, Belfast, Northern Ireland BT7 1NN, U.K.; 3 Analytical Instrumentation Facility, 6798 North Carolina State University, Raleigh, North Carolina 27695, United States; 4 Charles L. Brown Department of Electrical and Computer Engineering, 2358 University of Virginia, Charlottesville, Virginia 22904, United States; 5 SciTech Daresbury Campus, SuperSTEM Laboratory, Daresbury WA4 4AD, U.K.; 6 School of Chemical and Process Engineering, University of Leeds, Leeds LS2 9JT, U.K.

## Abstract

Hafnium oxide-based thin films, in particular hafnium zirconium oxide (HZO), have potential for applications in nonvolatile memory and energy harvesting. Atomic layer deposition (ALD) is the most widely used method for HZO deposition due to its precise thickness control and ability to provide conformal coverage. Previous studies have shown the effects of different metal precursors, oxidizer precursors, and process temperatures on the ferroelectric properties of HZO. However, no mechanism has been identified to describe the different phase stabilities as the metal precursor purge time varies. This study investigates how varying the metal precursor purge time during plasma-enhanced ALD (PE-ALD) influences the phases and properties of the HZO thin films. Grazing incidence X-ray diffraction, Fourier transform infrared spectroscopy, and scanning transmission electron microscopy are used to study the changes in phase of HZO with variation of the metal precursor purge time during the PE-ALD process. The phases observed are correlated with polarization and relative permittivity responses under an electric field, including wake-up and endurance effects. The resulting phases and properties are linked to changes in composition, as measured using time-of-flight secondary ion mass spectrometry and X-ray photoelectron spectroscopy. It is shown that short metal precursor purge times result in increased carbon and nitrogen impurities and stabilization of the antipolar *Pbca* phase. Long purge times lead to films comprising predominantly the ferroelectric *Pca2*_*1*_ phase.

## Graphical abstract

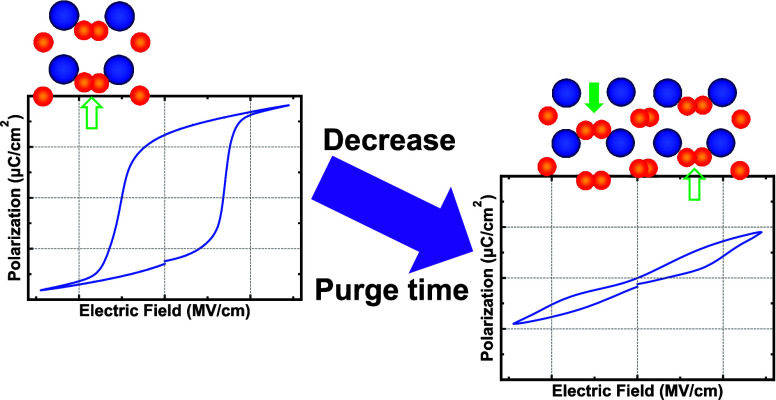

## Introduction

Ferroelectric thin films hold great promise toward advancing nonvolatile memories for computing applications.^1^ In comparison with competing technologies, ferroelectric-based devices have the advantages of low power consumption and a short access time. However, for conventional ferroelectric materials, including lead zirconate titanate, strontium bismuth tantalate, and barium titanate, it is challenging to achieve scalability and compatibility with complementary metal-oxide-semiconductor (CMOS) processes, particularly for back-end-of-line (BEOL) integration where process temperatures must be constrained. For example, many conventional ferroelectrics suffer from strong dimensional scaling effects, particularly for feature sizes of approximately 100 nm and less, and this is exacerbated in situations where epitaxy is not possible, such as in BEOL microelectronic applications.^2^ Ferroelectric fluorites, such as doped hafnium oxide (HfO_2_),^3^ overcome these integration challenges. Compared to conventional ferroelectric materials, HfO_2_-based ferroelectrics have chemical compatibility with traditional semiconductors, like silicon, exhibit ferroelectricity in nanometer-scale thicknesses with polycrystalline microstructures, and can be prepared as uniform layers over complex geometries by using atomic layer deposition (ALD).

HfO_2_ is currently used as a gate dielectric in most scaled CMOS technology nodes in an amorphous phase.^4^ Recently, however, there has been interest in applying it to nonvolatile memory,^3,5,6^ energy harvesting,^7^ infrared sensing,^8^ and negative differential capacitance field-effect transistors.^9^ In equilibrium at room temperature and pressure, HfO_2_ adopts a monoclinic phase (*m*-phase, space group *P*2_1_/*c*). This monoclinic phase is not suitable for use in charge-based nonvolatile memories because it lacks a spontaneous polarization. Ferroelectricity in HfO_2_-based materials results from a metastable orthorhombic phase (o-III phase, space group *Pca*2_1_) that is observed most commonly in polycrystalline films.^3,10^ Phase stability in the fluorites is affected by surface energy/area,^11–13^ stress,^14–16^ oxygen vacancy concentration,^17–21^ and doping.^22,23^ All of these factors have been indicated to stabilize the o-III phase in the HfO_2_ thin films. Among them, alloying with zirconium oxide (ZrO_2_) to form hafnium zirconium oxide (HZO) has been demonstrated to result in ferroelectric phase formation over a broad composition range with a maximum in performance typically observed in compositions near Hf_0.5_Zr_0.5_O_2–*x*_.^24^ In addition to compositional flexibility, HZO has several attractive properties, such as crystallization at low temperatures (i.e., <400 °C),^25^ thermodynamic compatibility of both HfO_2_ and ZrO_2_ with silicon,^26^ and having robust ALD processes. However, as with other doped HfO_2_ compositions, additional stabilizing factors are typically employed to maximize the o-III phase content and ferroelectric performance. For example, a capping electrode aids in stabilizing the ferroelectric phase over the monoclinic phase by preventing local out-of-plane deformations that accompany volume expansion as the monoclinic phase nucleates.^27,28^ In addition, processing parameters, such as annealing temperature, annealing time, and cooling rate, have been indicated to improve the ferroelectric properties of HZO thin films and the stabilization of the o-III phase.^29^

As oxygen vacancy concentrations and microstructure are process dependent, deposition of HZO using different techniques affects the resulting phases and performance. ALD is the most common technique because of its superior thickness control, conformality, and relatively low process temperatures (e.g., <330 °C).^30^ There has been prior research on the effects of different metal precursors, oxidant precursors, and process temperature on the ferroelectric properties of HZO.^31^ With respect to oxidant precursors, ALD can be divided into thermal atomic layer deposition (TH-ALD), which commonly uses H_2_O, H_2_O_2_, or O_3_, and plasma-enhanced atomic layer deposition (PE-ALD), which uses an oxygen plasma. As examples of process dependence on properties, the use of tetrakis-dimethylamido-hafnium (TDMA-Hf) and tetrakis-dimethylamido-zirconium (TDMA-Zr) improved the quality of HZO films by adopting O_3_ as the oxidant instead of H_2_O due to a lower carbon impurity concentration.^32–34^ Additionally, it was demonstrated that HZO prepared using PE-ALD exhibited greater ferroelectric o-phase content.^35^ For a given set of precursors (tetrakis-ethylmethylamido-hafnium (TEMA-Hf) and tetrakis-ethylmethylamido-zirconium (TEMA-Zr) with oxygen plasma), Mittmann et al. showed that increasing the metal precursor purge time in between ALD pulses during the ALD processing of HZO results in a reduction of the monoclinic phase, an increase in o-III phase, and an increase in remanent polarization (*P*_r_).^36^ However, the mechanism that drove the different phase stabilities by changing the purge time was not identified.

In this report, the dependence of TEMA-Hf and TEMA-Zr precursor purge time during PE-ALD processing of nominally 10 nm thick HZO films on the resulting phases and electrical performance is studied. The films were prepared with precursor purge times varying from 3 to 90 s. Phases present after the crystallization annealing were studied using grazing incidence X-ray diffraction (GIXRD), Fourier transform infrared spectroscopy (FTIR), high-angle annular dark-field scanning transmission electron microscopy (HAADF-STEM), and differential phase contrast scanning transmission electron microscopy (DPC-STEM). Chemical composition and impurities were measured by using X-ray photoelectron spectroscopy (XPS) and time-of-flight secondary ion mass spectrometry (ToF-SIMS). Capacitor structures were prepared, and electrical properties of relative permittivity, polarization response, wake-up effects, and endurance were studied. The results show that chemical impurities, specifically carbon and nitrogen, are concomitant with a stabilization of a nonferroelectric *Pbca* phase (o-I phase) that exhibits an antiferroelectric response. Increasing the metal precursor purge time reduces carbon and nitrogen impurity levels and results in larger fractions of the o-III phase and higher remanent polarizations.

## Experimental Method

### Sample Preparation

Pt/W/HZO/W/Si capacitor devices were prepared as follows: 50 nm of a tungsten bottom electrode was deposited on a (001)-oriented silicon substrate by DC magnetron sputtering within a custom high-vacuum instrument. The tungsten was sputtered from a Meivac MAK 50.4 mm diameter magnetically balanced sputter gun oriented at a 45° angle with respect to the substrate surface and a distance of 8 cm. A 3.3 W/cm^2^ power density and 5.0 mTorr argon background gas were used. The 10 nm thick HZO film was prepared by PE-ALD within an Oxford FlexAL II system. The metal precursors were TEMA-Hf and TEMA-Zr and were introduced from bubbled sources held at 70 and 85 °C, respectively. The precursor manifold and lines into the chamber were held at 120 and 140 °C, respectively, to prevent precursor condensation. The table temperature within the ALD reactor was set at 260 °C. Argon was present in the reactor during the metal precursor dosing steps at a pressure of 80 mTorr. The precursor purge was conducted at a pressure of 80 mTorr comprising argon gas for durations ranging from 3 to 90 s (3, 6, 15, 30, 60, and 90 s were studied). An oxygen plasma was used as the oxygen precursor at powers and durations of 250 W and 3 s and 300 W and 6 s for the Hf and Zr layers, respectively. A cycle of 3:2 Hf:Zr doses was used to prepare an approximately Hf_0.5_Zr_0.5_O_2–*x*_ stoichiometry. In each cycle, TEMA-Hf was dosed for 1.0 s, and TEMA-Zr was dosed for 1.5 s. The independent growth rates for HfO_2_ and ZrO_2_ using these doses are 1.17 and 1.34 Å/cycle, respectively. Seventeen super cycles were deposited to result in approximately 10 nm thick films. All other conditions were kept constant. Next, 20 nm of tungsten as a top electrode/capping layer was deposited by using the same conditions as for the bottom electrode; only the deposition time was reduced. All samples were rapid thermal annealed using an Allwin21 AccuThermo AW 610 instrument at 600 °C for 30 s with a 1 atm pressure, N_2_ ambient, and a heating ramp rate of 50 °C/s. Films that were characterized for phase via X-ray diffraction were kept with a continuous tungsten top layer. For electrically characterized devices, 50 nm of platinum was deposited through a shadow mask to create a capacitor structure. Remaining tungsten was then etched from the field using a commercial tungsten etchant (Sigma-Aldrich Tungsten Etchant, KOH: ≤10%, *x*-C_6_N_6_FeK_3_: 10 ≤ *x* < 20%). Electrode diameters used for measurements were approximately 100 μm, and their areas were verified by optical microscopy.

### Characterization

GIXRD and X-ray reflectivity (XRR) was performed on samples without platinum contacts using a Rigaku SmartLab X-ray diffractometer with Cu Kα radiation. GIXRD was collected with a fixed incident angle (ω) of 1.0°. A 2θ range from 26 to 33° was chosen because it contains the high-intensity reflections of the monoclinic, tetragonal (space group *P*4_2_/*nmc*), o-I, and o-III phases. The XRR patterns were fit using GSAS-II software.^37^ 2D X-ray diffraction patterns were collected using a Bruker D8 Venture diffractometer with a Photon III detector, an ω incidence angle of 18°, and an Incoatec IμS 3.0 Cu Kα radiation source. MgO powder was adhered to the sample surface and used as a stress-free height alignment standard for the 2D measurement. The 2D detector patterns were analyzed using the pyFAI azimuthal integration package.^38^ To calculate the stress state of each film, sin^2^(ψ) analysis was performed on the crystallized films using intensity profiles extracted at ψ angles between 0 and 45° relative to the film surface normal. An elastic modulus of 209 GPa was used for HZO.^39^ FTIR was performed with a Bruker Invenio-S FTIR spectrometer with single-reflection diamond attenuated total reflectance (ATR) attachment. A wavenumber range from 400 to 900 cm^–1^ was chosen because it contains the high-intensity absorbances of the monoclinic, tetragonal, antipolar orthorhombic o-I, and polar orthorhombic o-III phases.^40,41^ A spectral resolution of 4 cm^–1^ was used for all measurements. Each measurement was baseline corrected using OPUS 8.0 software. ToF-SIMS was performed by using an IONTOF TOF-SIMS 5 instrument. Each measurement was obtained using a dual-beam analysis setup with the following conditions: a 170 μm × 170 μm crater was created using a 3 keV cesium (Cs^+^) ion beam at a current of 15 nA, and the central 50 μm × 50 μm region of the sputtered crater was analyzed using a 0.3 pA 25 keV Bi^3+^ primary ion beam. Negative secondary ion mass spectra were obtained focusing on the following species: C^–^, CN^–^, O_2_^–^, SiO_2_^–^, WO_3_^–^, HfO_2_^–^, and ZrO_2_^–^. XPS spectra were collected with a Scienta Omicron XM1200 instrument with monochromated Al Kα X-rays (1486.7 eV). A survey scan was captured between 0 and 1300 eV using a pass energy of 200 eV. The Zr 3d, C 1s, O 1s, and Hf 4f peaks were identified, and high-resolution scans were subsequently performed for each peak using a pass energy of 50 eV. The sensitivity factors used for quantification were Hf 4f: 2.221, Zr 3d: 2.216, O 1s:0.711, and C 1s: 0.296. Using KolXPD software,^42^ the high-resolution scans were fitted, quantified, and analyzed. A focused ion beam (FIB) was used to prepare cross-sectional lamellae for transmission electron microscopy. A Tescan focused ion beam-secondary electron microscope (SEM) Lyra 3 was used. FIB milling was performed with gallium ions at accelerating voltages of 30 kV (rough milling) and 5, 2, and 1 kV (fine polishing). Scanning transmission electron microscopy (STEM) measurements were acquired on a Nion UltraSTEM 100 operated at 100 kV and a ThermoFisher Scientific Talos F200X instrument operated at a 200 kV accelerating voltage. The Nion UltraSTEM 100 is equipped with a fifth-order probe aberration corrector, which enables configurations of the probe-forming optics to achieve a probe size of <1 Å at the accelerating voltage of 100 kV, with a convergence semiangle of 30 mrad and a probe current of ∼30 pA. The collection semiangle range for all HAADF-STEM images was 89–195 mrad. Image stacks (30 or more images, each with a size of 1024 × 1024 pixels) were acquired using a short dwell time (5–10 μs per pixel) to minimize drift and beam damage. A high signal-to-noise ratio image was then reconstructed from these stacks after rigid and/or nonrigid registration. For DPC imaging, a segmented detector (ThermoFisher) was used to measure deflection of the transmitted electron beam. The contrast mechanism results from local electromagnetic fields in the specimen and/or variation in crystallographic orientation deflecting the beam. Atomic force microscope (AFM) topography was measured using an Oxford Instruments Asylum Research Cypher-S instrument in AC mode at resonance with a BudgetSensors Tap300Al-G cantilever. The Hilliard circular intercept method from ASTM E112-10 was used to estimate the mean intercept length, which approximates grain size, by using ImageJ (ver. 1.53K).^43^

Electrical properties, including polarization hysteresis (polarization versus electric field, *P*(*E*)), positive up–negative down (PUND) polarization, and capacitance–voltage (*C*–*V*), were measured on each sample. *P*(*E*) and PUND measurements were performed by using a Radiant Technologies Precision LC II Ferroelectric Tester. Nested *P*(*E*) measurements were performed by using a maximum applied field ranging from 0.5 to 2.5 MV/cm with a period of 1 ms. PUND measurements utilized a 2.5 MV/cm amplitude square pulse with a width of 1 ms and a pulse delay of 100 ms. *C*–*V* measurements spanned ± 2.5 MV/cm with a 10 kHz, 50 mV oscillator and were carried out with a Keysight E4980A Precision LCR Meter. On these samples, measurements were taken before and after 5 × 10^3^ cycles of a 2.0 MV/cm 1 kHz square wave for ferroelectric wake-up. For the endurance test, the minimum sufficient pulse duration to achieve complete polarization switching was determined using the method described by Lenox et al.^44^ A customized bipolar pulse waveform was used with the following parameters: 10 kHz frequency with 2.0 MV/cm, 2 μs pulse duration, and 20 ns rise and fall times. During the endurance tests, all measurements started on pristine unpoled capacitors. A Keysight 33511B waveform generator was employed as an external input to facilitate the cycling of all samples using the custom bipolar waveform. Properties were measured at decade intervals and, near the failure point, at linear intervals to focus on the leakage current as the capacitors degraded at high cycle counts.

## Results and Discussion

The GIXRD patterns in Figure 1a show a single peak positioned at approximately 30.6 degrees in 2θ. This peak is characteristic of the (111) plane of the ferroelectric polar orthorhombic o-III phase, (211) plane of the antipolar orthorhombic o-I phase, and/or (101) plane of the tetragonal phase. The peaks of the three different crystal structures are indistinguishable from each other due to the similarity in *d*-spacing, the wide peak widths associated with the fine crystallite sizes, and uncertainty in reference peak positions owing to biaxial strain. There are no peaks of the nonferroelectric monoclinic phase, such as the (1̅11) and (111) reflections. Combined, these GIXRD data suggest that regardless of the metal precursor purge duration used, all films comprise metastable phases.

**Figure 1:**
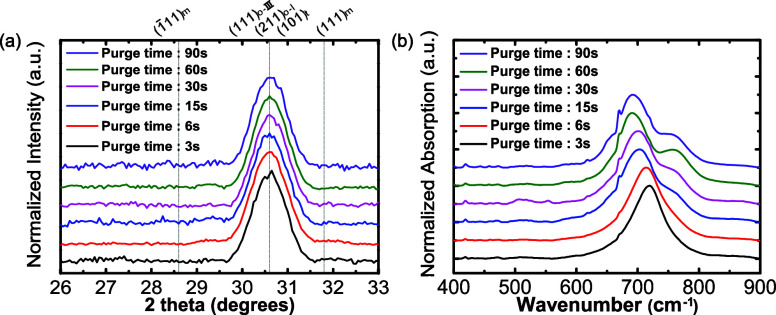
(a) GIXRD patterns of HZO thin films with different metal precursor purge times (3, 6, 15, 30, 60, and 90 s). The dashed lines indicate the position of monoclinic (m), polar orthorhombic (o-III), antipolar orthorhombic (o-I), and tetragonal (t) phases. Owing to uncertainty in the unstrained peak positions for the three metastable phases, they are denoted as being in the same location. (b) Normalized FTIR-ATR absorption spectra of the HZO thin films with varying precursor purge times. The narrow absorption at 670 cm^–1^ is a measurement artifact.

To better identify the possible phases present, IR absorption spectra were measured. The FTIR-ATR spectra for each film are shown in Figure 1b. All collected spectra were normalized to their maximum signal within the 400–900 cm^–1^ range. When the HZO thin films were prepared with a purge time of 3 s, the major peak was located at ∼720 cm^–1^. As the metal precursor purge time increased, the major absorbance peak shifted to lower energies. When the purge time was 6 s, the peak is approximately 713 cm^–1^, and when the metal precursor purge time was 15, 30, 60, and 90 s, the peaks are 704, 700, 691, and 692 cm^–1^, respectively. Furthermore, a second peak was observed to emerge starting from a purge time of 15 s. The purge times of 15, 30, 60, and 90 s all show the second peak at approximately 760 cm^–1^. As mentioned in discussing the GIXRD data, all samples have the same reflection at a 2θ angle of 30.6°; however, in Figure 1b, the FTIR-ATR spectra red shift as the metal precursor purge time increases and a new peak emerges. Prior work has shown that the polar orthorhombic, antipolar orthorhombic, and monoclinic phases have different spectral shapes when measured by synchrotron infrared nano spectroscopy (SINS/nano-FTIR).^40^ The ferroelectric o-III phase of HZO shows prominent peaks at 670 and 760 cm^–1^ in SINS measurements.^45^ The antipolar o-I phase of HZO has no feature at 760 cm^–1^ and a narrower and slightly blue-shifted strong absorption near 670 cm^–1^. The films in those studies did not contain the tetragonal phase in identifiable quantities as assessed by STEM imaging, and theory suggests no strong tetragonal infrared absorption features above 600 cm^–1^.^11,41^ Using the theory and prior experimental results as a guide, the FTIR-ATR results in this study show that reducing the metal precursor purge time results in a decrease in intensity and, ultimately, a disappearance of the 760 cm^–1^ absorption and a blue shift of the strongest absorption feature. This suggests that films prepared with a long precursor purge time crystallize into the ferroelectric o-III phase, while the short duration precursor purges result in crystallization into another phase, which is likely to be the antipolar o-I phase. It is noted that the GIXRD data do not show evidence of the monoclinic phase, so the absorption at 760 cm^–1^ cannot be assigned to that phase, and the phase of the long purge time samples is o-III. Furthermore, the sharp absorption at 670 cm^–1^ in all the spectra in this work is too narrow to be attributed to the HZO films and is a measurement artifact.

Figure 2 shows the polarization responses for films prepared with different metal precursor purge times (90, 30, and 3 s). Corresponding data for the 60, 15, and 6 s precursor purge times can be found in the Supporting Information, Figure S1. Figure 2a,c,e shows the pristine *P*(*E*) responses, while Figure 2b,d,f shows the responses after wake-up field cycling with 5 × 10^3^ 2 MV/cm square waves. Figure 2a,b, which is for the 90 s purge time sample, shows that the hysteresis response is well saturated with a minimal degree of pinching after wake-up field cycling. This is similar to many other reports of ferroelectricity in HZO.^32–36^ Compared to the sample processed with a purge time of 90 s, the hysteresis loops measured on 30 s purge time devices are more pinched (Figure 2c,d). In addition, as shown in Figure 2e,f, the 3 s purge time devices are significantly more pinched, the saturation polarization is less than 20 μC/cm^2^, and the remanent polarization converges toward zero. The antiferroelectric-like responses suggest that the HZO thin films prepared with low purge time contain different phases, such as the tetragonal phase or antipolar o-I phase, or have severely pinned domain walls. However, according to the FTIR-ATR results (Figure 1b), there is a strong absorption feature above 600 cm^–1^, suggesting that these films have low volume fractions of tetragonal phase. Furthermore, the FTIR results clearly show that this film does not contain large fractions of the o-III phase in the as-prepared state, so the pinched *P*(*E*) response is not likely to be due to pinned domain walls, but rather, it can be expected that this antiferroelectric property comes from the antipolar o-I phase. Comparing the remaining *P*(*E*) responses (Figure S1), it can be observed that as the metal precursor purge time decreases, the saturation and remanent polarizations also decrease.

**Figure 2:**
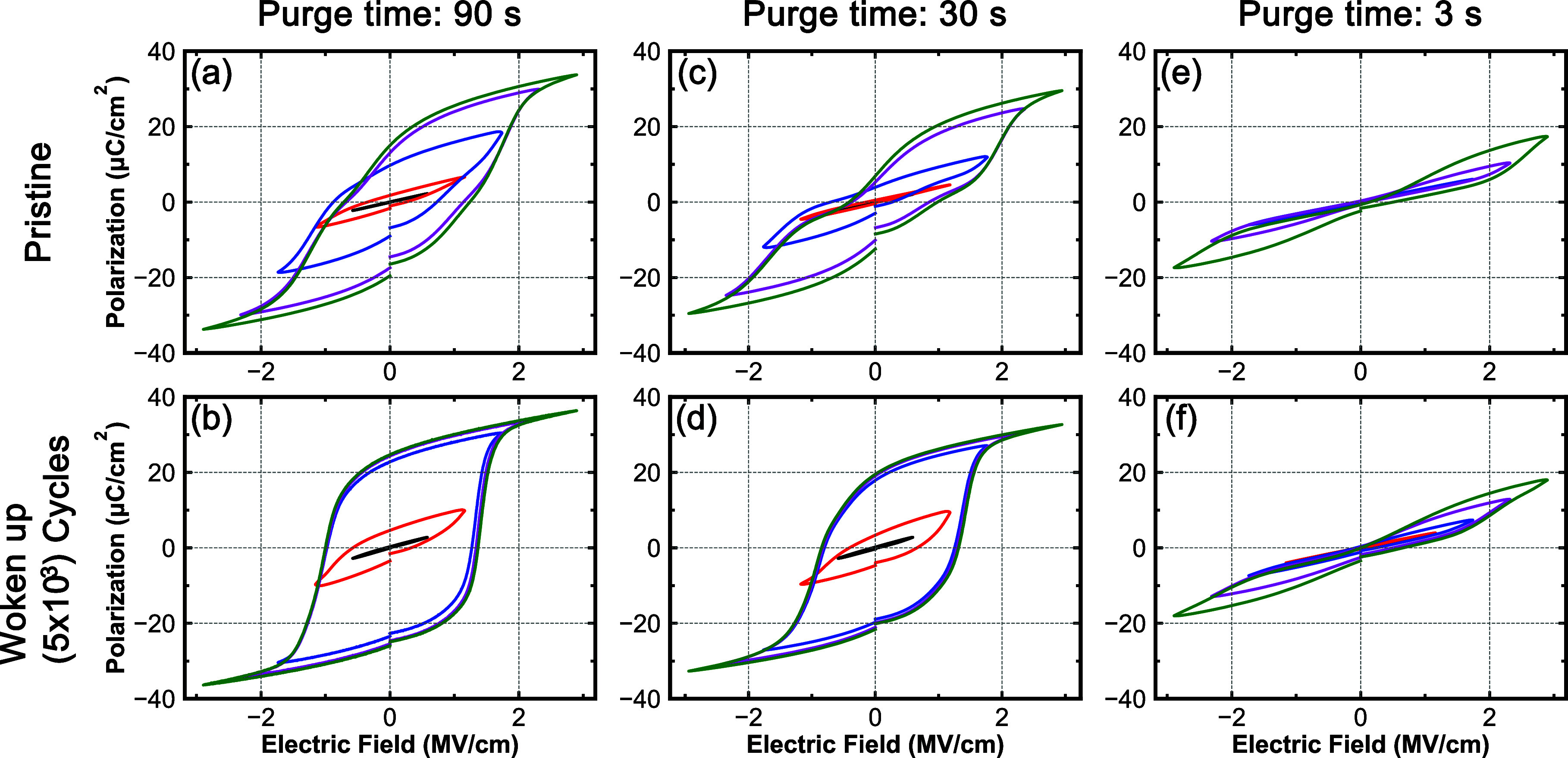
Top row shows nested *P*(*E*) responses measured on pristine HZO devices prepared with (a) 90 s, (c) 30, and (e) 3 s purge times. The bottom row shows nested awoken *P*(*E*) responses (5 × 10^3^ cycles) measured on HZO devices prepared with (b) 90 s, (d) 30 s, and (f) 3 s purge times.

Figure 3 shows the *P*(*E*) hysteresis and switching current densities (*J*(*E*)) at decade spaced cycling intervals from pristine (10^0^) to 10^9^ cycles for the HZO devices with various metal organic precursor purge times (90, 30, and 3 s). In addition, each device was measured at linearly spaced cycling intervals from 10^7^ cycles and beyond to monitor the progression of polarization and leakage currents. *P*(*E*) and *J*(*E*) results for purge times of 60, 15, and 6 s of HZO devices are included in the Supporting Information in Figure S2. For the 90 s purge time devices, *P*(*E*) and *J*(*E*) behaviors are consistent with a ferroelectric response, as shown in Figure 3a,b. While clearly ferroelectric in the pristine condition, the *P*_r_ increases steadily and continuously due to wake-up, which can be attributed to a phase transformation from nonferroelectric phases to the ferroelectric phase and ferroelastic switching.^45–49^ No significant degradation is observed up to 6 × 10^7^ cycles, where clear polarization saturation is observed. In Figure 3b, the current density, *J*(*E*), is shown from pristine to 6 × 10^7^ cycles. The peak at approximately 1.0 MV/cm corresponds to the coercive fields. These peaks show an increase in the maximum current density and a decrease in the width, which is consistent with the narrowing of the field distribution required to switch domains. However, the device could not be measured after 6 × 10^7^ field cycles because the HZO device broke down, though there was no significant degradation in leakage current prior to the breakdown event. For the 30 s purge time HZO devices, as shown in Figure 3c,d, the behavior changes from containing an antiferroelectric component to largely ferroelectric with field cycling. Reasonable *P*(*E*) polarization saturation is observed at the maximum applied field when field cycling is up to 2 × 10^8^ cycling; however, the device broke down after this point, again with a minimal increase in leakage prior to breakdown. Lastly, for the purge time of 3 s, as shown in Figure 3e,f, antiferroelectric behavior is seen for all cycling conditions with limited wake-up behavior and minimal *P*_r_ variation. No fatigue was seen through 10^9^ cycles, as the maximum polarization value did not exhibit any decrease. The *J*(*E*) response shown in Figure 3f reveals four switching peaks. The positive current peak is located at ∼2.1 MV/cm. This *J*(*E*) response is typical of those of antiferroelectric materials and is consistent with the antipolar o-I phase. Thus, it can be considered to have antiferroelectricity when the metal precursor purge time used to prepare HZO devices is 3 s. This device did not break down to 10^9^ field cycles, at which point the measurement was stopped. Several studies have reported that antiferroelectric HZO and ZrO_2_ may exhibit a high endurance of 10^12^ cycles without fatigue. However, these devices comprise HZO compositions that are zirconium rich, which has been reported to stabilize the tetragonal phase,^50–52^ though recent reports confirm the presence of the o-I phase in antiferroelectric, high Zr-containing HZO with long endurance.^53,54^

**Figure 3:**
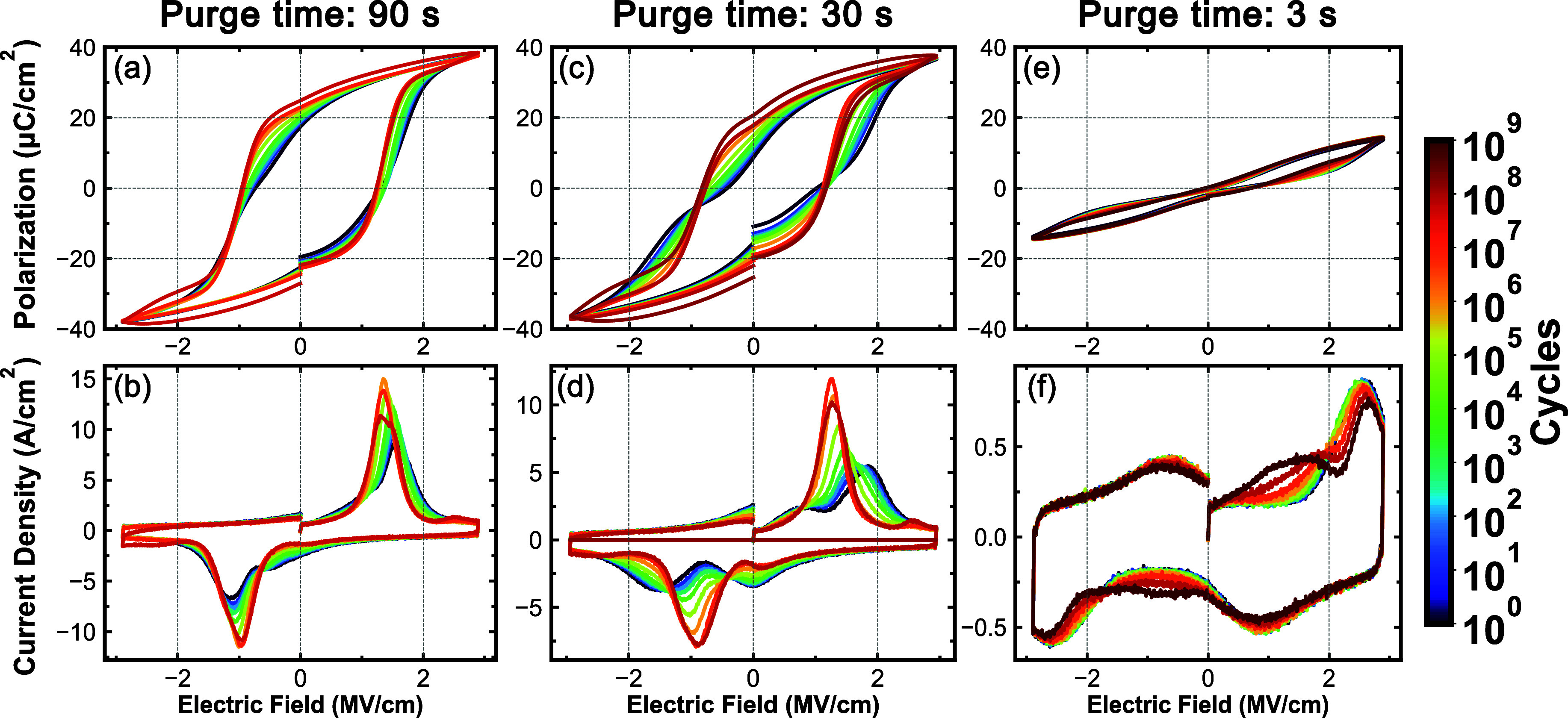
Top row shows decade fatigue cycling *P*(*E*) hysteresis loops from 1 to 10^9^ cycles on HZO devices with (a) 90, (c) 30, and (e) 3 s purge times. The bottom row shows decade fatigue cycling current loops from 1 to 10^9^ cycles on HZO devices with (b) 90 s, (d) 30, and (f) 3 s purge times.

PUND measurements improve accuracy in remanent polarization measurements by subtracting leakage current and linear dielectric contributions to the charge recorded in a dielectric displacement measurement.^55^ PUND measurements were performed for pristine and awakened capacitors with the results shown in Figure 4a. Confirming the polarization response shown in Figure 2, at 90 s of purge time, the remanent polarization after wake-up field cycling is greater than 20 μC/cm^2^, typical of ferroelectric HZO. As the purge time decreased, the remanent polarization decreased, and at a purge time of less than 6 s, the remanent polarization converged to zero. In addition, PUND was performed during the endurance test, as shown in Figure 4b. When the metal precursor purge time of the HZO devices was low (i.e., 3 and 6 s), the remanent polarization remained almost constant, albeit with values less than 5 μC/cm^2^, without fatigue up to 10^9^ cycles, consistent with the *P*(*E*) responses in Figure 3. However, HZO devices exhibited wake-up and fatigue when the purge time was 15 s followed by failure at 5 × 10^8^ cycles. Longer purge durations displayed wake-up but did not fatigue prior to dielectric breakdown. Dielectric breakdown was observed after, 2 × 10^8^, 10^8^, and 6 × 10^7^ for purge times of 15, 30, 60, and 90 s, respectively. The sudden breakdown may be related to oxygen vacancies and conductive filament formation,^56^ but more research is necessary to definitively identify the failure mechanism.

**Figure 4:**
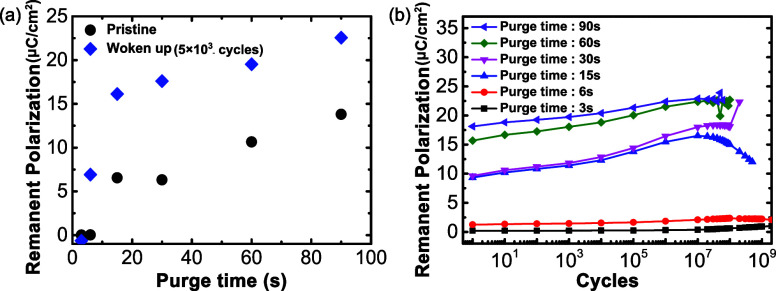
(a) PUND-measured remanent polarizations before (black) and after (blue) 5 × 10^3^ wake-up field cycles for the same HZO devices with varied metal precursor purge times. (b) PUND-measured remanent polarization during fatigue cycling of from 10 to 10^9^ cycles on HZO devices with varied metal precursor purge times.

The corresponding *C*–*V* measurements are provided in Figure 5. The *C*–*V* measurements of the 90 s purge time devices are shown in Figure 5a. There are two peaks in the relative permittivity, indicating a ferroelectric response. The 30 s purge time devices, as shown in Figure 5b, reveal a mixed behavior of ferroelectric and antiferroelectric responses, with two major peaks and two minor peaks. In Figure 5c, the response of the 3 s purge time devices shows four peaks, which are characteristic of antiferroelectricity. As the relative permittivity is proportional to the derivative of the dielectric displacement, these behaviors are consistent with the polarization hysteresis shown in Figure 2. Moreover, Figure 5d shows the variation of the relative permittivity at a high electric field (−2.5 MV/cm) as a function of the metal precursor purge time. The relative permittivity is 26.8 when the purge time is 90 s, and they are 27.8 and 32.1 when the purge times are 30 and 3 s, respectively. The relative permittivity tends to increase as the purge time decreases, but the differences are relatively small. According to prior reports, the tetragonal phase typically possesses a significantly higher relative permittivity than the polar o-III phase, with values up to 70 reported.^57^ The relative permittivity of the o-I phase has been reported to be slightly higher than that of the o-III phase.^40^ Based on this data, at a purge time of 3 s, HZO devices are more consistent with the o-I phase than with the tetragonal phase.^40,45^ *C*–*V* results for the purge times of 6, 15, and 60 s are included in Figure S3.

**Figure 5:**
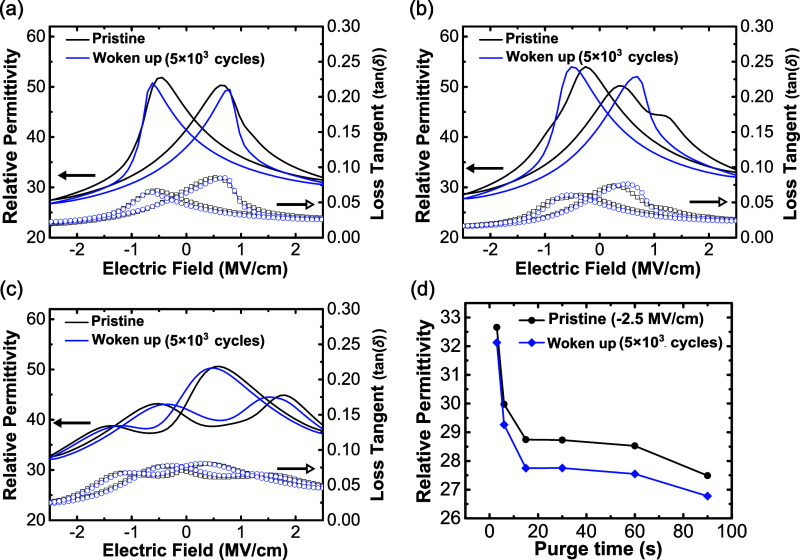
10 kHz relative permittivity (black and blue solid lines, left axis) and associated loss tangents (black and blue square points, right axis) measured on HZO devices with (a) 90, (b) 30, and (c) 3 s purge times and (d) relative permittivity of HZO devices with different metal precursor purge times in the pristine (black) and awakened (blue) states.

HAADF-STEM studies were performed to verify the phase assignments in the 3 and 90 s purge time samples. Figure 6a displays a HAADF-STEM image of a grain in the HZO thin film grown with a 3 s purge time, while Figure 6b displays an image of a grain in the 90 s purge time film. A fast Fourier transform (FFT) was performed on each image to aid in the phase identification. The resulting diffraction patterns for the 3 and 90 s purge time samples can be indexed to orthorhombic phases. Differential phase contrast (DPC) imaging was employed to provide a larger-scale view of the impact of the purge time on the resultant phase formation in HZO thin films. Recently, ferroelectric domains have been successfully imaged using DPC-STEM.^58–60^ The technique involves a segmented detector that measures deflections of the electron beam due to electromagnetic fields or differences in crystallographic orientation in the TEM specimen. While crystallographic orientation influences the DPC signal, as evidenced by the varied colors within the tungsten bottom electrode layers in Figure 6c,d, DPC-STEM is also an effective method for visualizing the polarization direction within ferroelectric domains. In Figure 6c,d, a color wheel indicates the polarization direction within the HZO thin film with intensity representing the magnitude of polarization. DPC-STEM imaging of the 3 s purge time compared to the 90 s purge time thin films reveals differences. In Figure 6c, the 3 s purge time sample, observations include: (i) significant grain size variation (grain 1 being >5 times larger than grain 2 (∼5 nm)) and (ii) limited DPC-STEM signal, signifying few ferroelectric grains, where grains 1 (green), 2 (red), and 3 (purple) show different polarization orientations, while other grains show no intensity. This lack of intensity could be due to a polar orientation being parallel to the electron beam or the grain being nonpolar (e.g., in the o-I phase where the alternating polarization directions lead to centrosymmetricity to give a lack of DPC signal). In contrast, Figure 6d from the 90 s purge time film shows (i) more uniform grain sizes, (ii) a greater number of ferroelectric grains (e.g., grains 1–6 with different polarization orientations), and (iii) varying polarization intensities, even among grains with similar orientations. DPC-STEM imaging corroborates the *P*(*E*) response, confirming that the HZO film grown with the longer purge time (90 s) demonstrates stronger ferroelectric behavior, whereas the HZO film grown with the shortest purge time (3 s) exhibits more pronounced nonpolar characteristics at low fields. These results are consistent with the phase assignments from GIXRD and FTIR-ATR.

**Figure 6:**
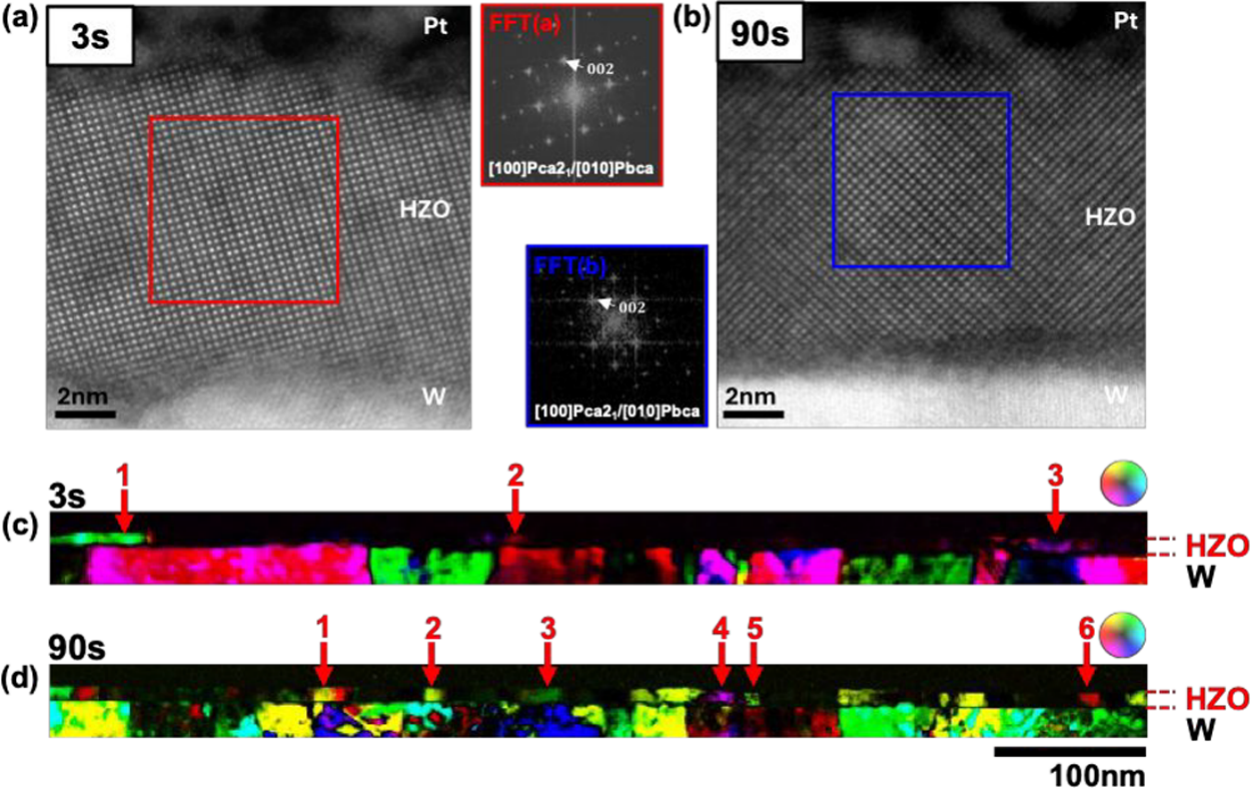
HAADF-STEM images of HZO grown with (a) 3 s purge time and (b) 90 s purge time. The red and blue squares denote the regions where the respective FFTs were measured. Panels (c) and (d) show the DPC-STEM images of HZO grown with (c) 3 s purge time and (d) 90 s purge time. The brightness of panels (c) and (d) has been increased by 30% to enhance visibility. The numbers and arrows highlight specific grains discussed in the text. The color wheel denotes the polarization direction in each region. Color in the tungsten grains is due to differing crystallographic orientations.

To understand the origin of the crystal structure change in HZO according to the metal precursor purge time, several additional known phase stabilizing parameters were measured: film stress, grain size, composition, and impurity content. 2D X-ray diffraction was employed with sin^2^ψ stress analysis, as shown in the Supporting Information, Figure S4. Based on the stress measurements, all HZO thin films are in a tensile stress state between 3.0 and 3.9 GPa after processing, which is reported to affect orthorhombic phase stabilization and the domain structure.^27,61,62^ There is no apparent trend in biaxial stress with different metal purge times within the measurement error. The grain size in the HZO thin film was quantified via AFM topography images (Figure S5) to explore possible grain size effects that may drive phase stabilization. As shown in Figure 7, the average grain size of HZO decreased as the purge time increased from an average lateral intercept distance of 18.2 ± 0.1 to 16.2 ± 0.1 nm for the 3 and 90 s purge time samples, respectively. Prior computational research suggested that the tetragonal phase, and thus a material that exhibits an antiferroelectric-like response, would be favored as the grain size is reduced.^63^ The opposite was observed here, with the long purge time films possessing the smallest average grain sizes and the strongest ferroelectric response. Therefore, neither stress nor grain size effects appear to be responsible for the phase differences observed in these films.

**Figure 7:**
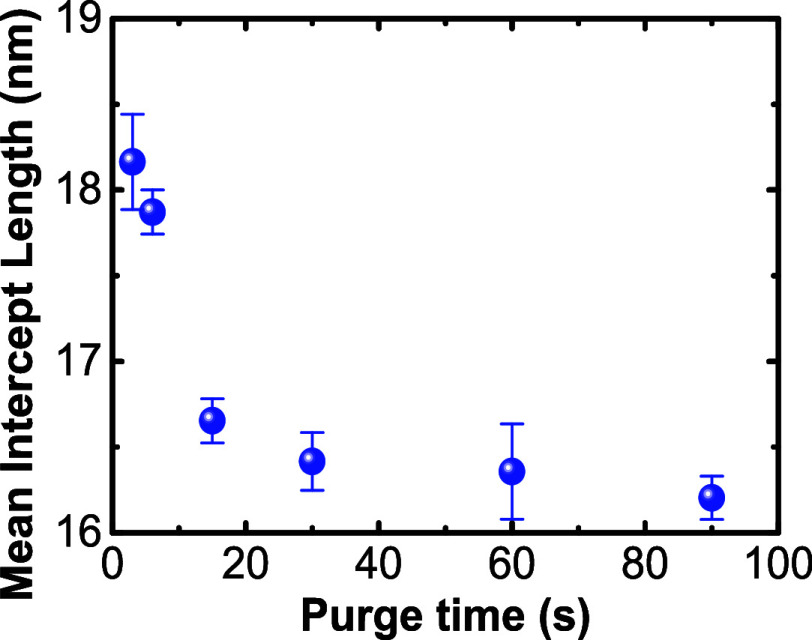
Mean intercept length (grain size) calculated by using the line-intercept method on the AFM images with error bars representing 95% confidence intervals.

Larger antiferroelectric-like responses are typically observed in HZO when the composition is ZrO_2_-rich.^24^ To explore whether the different precursor purge durations were affecting the composition, XPS was performed on the bare HZO surfaces after removal of the tungsten electrode. The Hf 4f and Zr 3d peaks and their respective fits are shown in the Supporting Information, Figures S9 and S10. The results are shown in Figure 8a. Here, it can be observed that increasing the precursor purge time results in a decrease in the hafnium content of the films. The Hf:Zr ratio changes from 79:100 for the 3 s purge time to 74:100 for the 90 s purge time. These correspond to compositions of Hf_0.45_Zr_0.55_O_2–*x*_ and Hf_0.43_Zr_0.57_O_2–*x*_ for the 3 and 90 s purge conditions, respectively. While TEMA-Zr has a higher vapor pressure than TEMA-Hf at a given temperature,^64^ it is observed that the hafnium concentration decreases with precursor purge time. It is hypothesized that the Hf:Zr ratio change with varying purge times is related to differences in the sticking coefficients of the Hf and Zr precursors and not their vapor pressures. The increased zirconium content in the longer precursor purge times is inconsistent with the stronger ferroelectric responses in these films, as higher Zr contents typically lead to antiferroelectric responses.^24^ This suggests that the small change in Hf:Zr composition is not driving the different phase stabilities. The O:(Hf+Zr) ratios are relatively consistent within each processing condition (Figure 8b). However, small differences may arise from surface overlayers, such as adventitious carbon and absorbed OH, which can unevenly attenuate XPS signals depending on electron kinetic energy.^65^ This may lead to differences between samples and introduce error due to differing overlayer thickness, so the differences in stoichiometry should not be directly linked to processing alone, nor should this reported oxygen stoichiometry be considered as absolute.^66,67^

**Figure 8:**
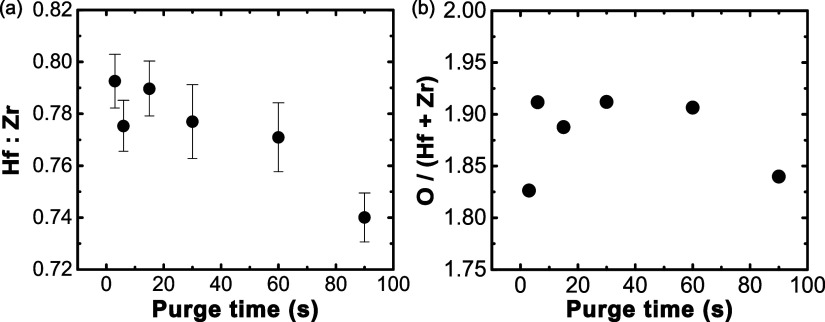
XPS-derived relative (a) Hf:Zr ratio and (b) O divided by (Hf+Zr) ratio with respect to metal precursor purge time.

Finally, the role of impurities caused by changing the metal precursor purge time during the PE-ALD process was investigated. In the ideal case, the organic ligand on the metal precursors such as TEMA-Hf and TEMA-Zr is eliminated through the oxidation and following purge process. Reducing the precursor purge time may result in residual impurities such as C, N, and H in the HZO due to unstable chemical reactions of residual metal precursors.^68,69^ To check for residual impurities in the HZO thin films, ToF-SIMS was performed through the W/HZO/W capacitors. While ToF-SIMS offers high sensitivity for detecting light elements, including hydrogen, accurate and quantitative analysis of hydrogen ions (H^+^) remains particularly challenging. In addition, a C^–^ signal was observed from the surface as well as within HZO layers. To compare the C content in the HZO layer, the C^–^ ion intensity was normalized to the HfO_2_^–^ + ZrO_2_^–^ intensity. Figure 9a and Figure S12a–c show the normalized carbon content in this region and the raw C^–^ signal, respectively. Note that the normalized C^–^:(HfO_2_^–^ + ZrO_2_^–^) ratio increases as the metal precursor purge time decreases. When the purge time is 3 s, carbon is present in higher concentrations than in the other samples. Reducing the metal precursor purge time increases the carbon impurities in the HZO thin film. Furthermore, elemental nitrogen exhibits an extremely low ion yield in SIMS due to its high ionization energy (14.5 eV), which greatly reduces the probability of ion formation during sputtering. As a result, nitrogen is typically detected indirectly via molecular fragments such as CN^–^, NO^–^, or NH^+^, which display significantly higher ionization efficiencies.^70^ In this study, the CN^–^ signal normalized to C^–^ and the raw CN^–^ signal was used to qualitatively assess nitrogen-related impurities, as shown in the ToF-SIMS depth profiles (Figure 9b and Figure S12d, respectively). As with nitrogen impurities, the CN^–^:C^–^ ratio increases as the metal precursor purge time decreases. Carbon and nitrogen have the highest concentration when the purge time is 3s and decrease as the purge time increases.

**Figure 9:**
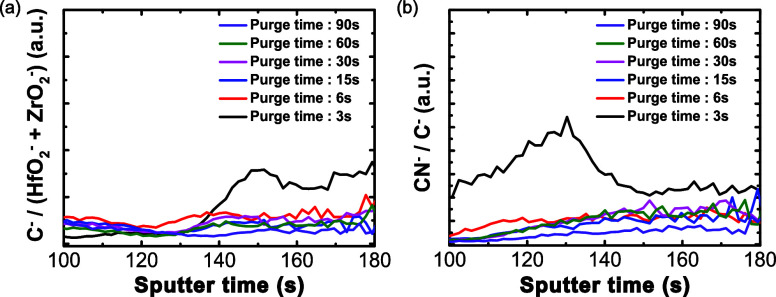
ToF-SIMS depth profiles of (a) C^–^ divided by (HfO_2_^–^ + ZrO_2_^–^) and (b) CN^–^ divided by C^–^ for the different metal precursor purge times.

Nitrogen incorporation in HfO_2_ and HZO has been previously shown to result in ferroelectric performance.^68,69,71^ However, excessive nitrogen incorporation leads to stabilization of metastable phases that are not ferroelectric,^71,72^ which is consistent with the observation in this work where the o-I phase has been shown to be predominant in films with the highest impurity levels.

The increase in carbon content when the metal precursor purge time was decreased suggests that carbon may also be correlated with changing the phase stability of HZO. The effect of carbon impurities on HZO was studied by several other research groups. Carbon incorporation into the interstitial site or oxygen substitutional positions of HfO_2_ could decrease the free energy of the tetragonal phase; thus, HfO_2_ might have a different phase stabilization.^73^ In addition, other studies have shown that HZO without carbon impurities has strong ferroelectricity with a high orthorhombic phase fraction. Carbon impurities in HZO have been suggested to result in the antiferroelectric-like double hysteresis generated from the field-induced phase transition between the tetragonal and orthorhombic phases.^74,75^ In this study, however, the tetragonal phase was not detected. Indeed, the antiferroelectric o-I was not largely discussed until 2022, when Cheng et al. observed it via STEM-annular bright-field (STEM-ABF),^50^ Kelley and Calderon et al. showed it DPC-STEM images,^76^ and Jaszewski et al. followed by showing antiferroelectric responses from HfO*_*x*_* films containing the o-I phase, as verified by DPC-STEM and SINS.^40^ This work shows that the polarization behavior of HZO becomes antiferroelectric as the metal precursor purge time decreases due to an increasing stabilization of the o-I phase and a concomitant increase in carbon and nitrogen impurities. These impurities appear to modify the free energy landscape in HZO and may lead to stabilization of the high-pressure o-I polymorph. The exact cause of the o-I phase stabilization by carbon and nitrogen impurities remains unclear and will need to be investigated further, likely driven by computation.

## Conclusions

This study investigated the effect of varying the metal precursor purge time during PE-ALD on the HZO capacitor properties. When the metal precursor purge time was reduced from 90 to 3 s, the electrical properties changed from ferroelectric to antiferroelectric. This change was supported by characteristics associated with antiferroelectricity, such as clear antiferroelectric polarization responses, an increased number of field cycles to fatigue, and a slight increase in relative permittivity. FTIR-ATR measurements support the idea that decreasing metal precursor purge time causes a change from the polar orthorhombic o-III phase to the antipolar orthorhombic o-I phase. This is consistent with data from HAADF-STEM and DPC-STEM. According to the ToF-SIMS results, it was found that reducing the metal precursor purge time during PE-ALD processing did not completely remove the chemical ligands, resulting in carbon and nitrogen impurities in the HZO film. This suggests that carbon and nitrogen impurities in HZO may cause a stabilization of the antipolar o-I phase. Therefore, this work demonstrates that phases in fluorite oxides are sensitive to impurities and that stable antiferroelectric responses can be achieved without intentionally modifying the material composition, such as using the Hf:Zr ratio to stabilize various phases.

## Supplementary Material



## Notes

Data showing polarization-electric field (*P*–*E*) measurements and fatigue analysis, dielectric measurements showing relative permittivity and loss tangent, thin film stress measured with the X-ray diffraction sin^2^ψ technique, atomic force microscope topography images, X-ray reflectivity patterns and fits, X-ray photoelectron spectroscopy data and fits, and raw secondary ion mass spectrometry data (PDF)

## †

U.S. Naval Research Laboratory, Washington, DC 20375, United States (S.S.F.)
