# Supplementary material for: Effect of Precursor Purge Time on Plasma-Enhanced Atomic Layer Deposition-Prepared Ferroelectric Hf0.5Zr0.5O2 Phase and Performance
Source: ACS Omega. 2025 May 14;10(20):20524–35. doi: 10.1021/acsomega.5c01112 (PMC12120649; doi:10.1021/acsomega.5c01112)
Supplement: Supplementary file 1 [file ao5c01112_si_001.pdf]

Effect of precursor purge time on plasma-enhanced atomic layer deposition-prepared ferroelectric $Hf_{0.5}Zr_{0.5}O_2$ phase and performance

Yong Kyu Choi,[1] *Kristina Holsgrove,*[2] *Andrea Watson,*[1] *Benjamin L. Aronson,*[1] *Megan K. Lenox,*[1] *Liron Shvilberg,*[1] *Chuanzhen Zhou,*[3] *Shelby S. Fields,*[1,†] *Shihao Wang,*[5,6] *Stephen J. McDonnell,*[1] *Amit Kumar,*[2] *and Jon F. Ihlefeld*[1,4,*]

[1] Department of Materials Science and Engineering, University of Virginia, Charlottesville, VA 22904, USA

[2] School of Mathematics and Physics, Queen's University Belfast, Belfast, Northern Ireland BT7 1NN, U.K

[3] Analytical Instrumentation Facility, North Carolina State University, Raleigh, NC 27695, USA

[4] Charles L. Brown Department of Electrical and Computer Engineering, University of Virginia, Charlottesville, VA 22904, USA

[5] SuperSTEM Laboratory, SciTech Daresbury Campus, Daresbury WA4 4AD, U.K.

[6] School of Chemical and Process Engineering, University of Leeds, Leeds LS2 9JT, U.K.

*Correspondence to: jihlefeld@virginia.edu

[†] Present address: U.S. Naval Research Laboratory, Washington, D.C. 20375, USA

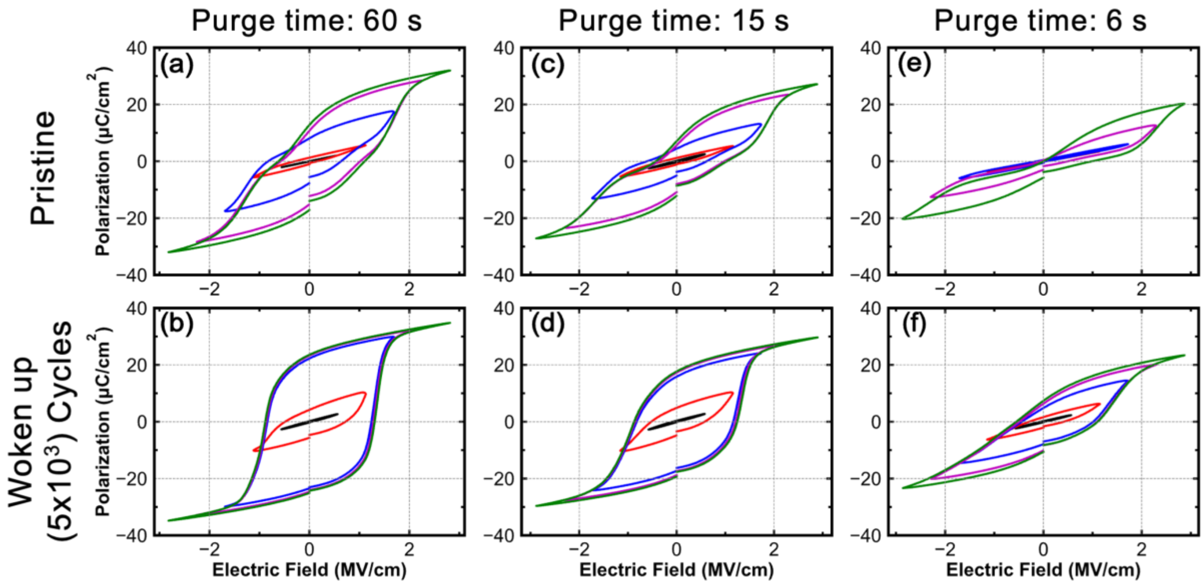

Figure S1. The top row shows nested $P(E)$ responses measured on pristine HZO devices prepared with (a) 60 s, (c) 15 s, and (e) 6 s purge times. The bottom row shows awoken $P(E)$ responses (5×10^3 cycles) measured on HZO devices prepared with (b) 60 s, (d) 15 s, and (f) 6 s purge times

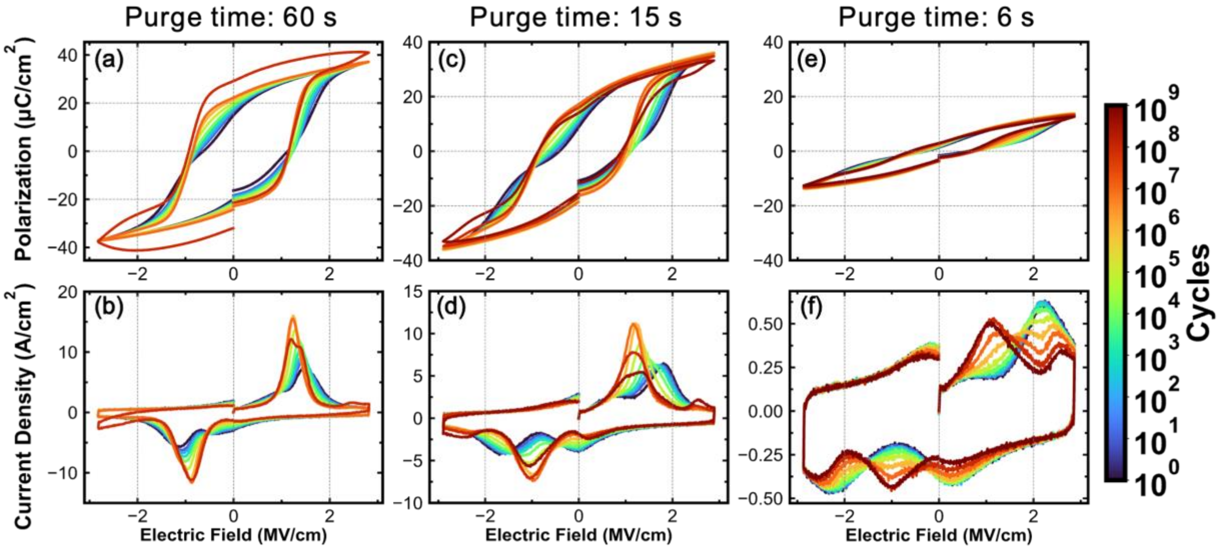

Figure S2. The top row shows decade fatigue cycling $P(E)$ hysteresis loop from 1-10^9 cycles on HZO devices with (a) 60 s purge time, (c) 15 s purge time, and (e) 6 s purge time. The bottom row shows decade fatigue cycling current loop from 1-10^9 cycles on HZO devices with (b) 60 s purge time, (d) 15 s purge time, and (f) 6 s purge time.

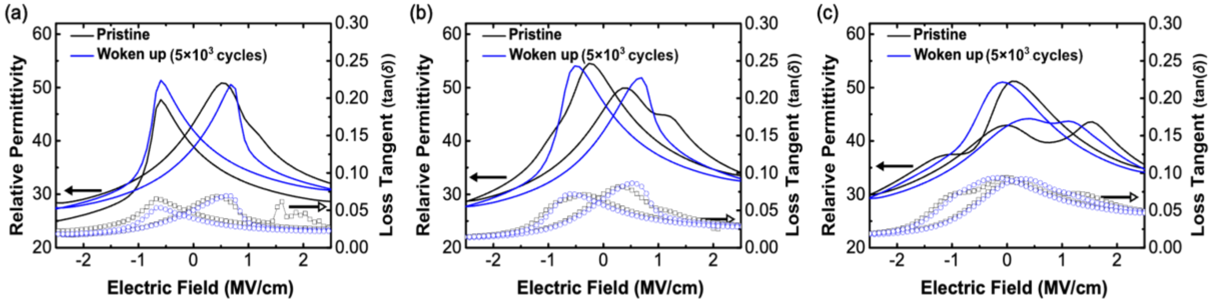

Figure S3. 10 kHz relative permittivity (black and blue solid lines, left axis) and associated loss tangents (black and blue square points, right axis) measured on HZO devices with (a) 60 s, (b) 15 s, and (c) 6 s purge times

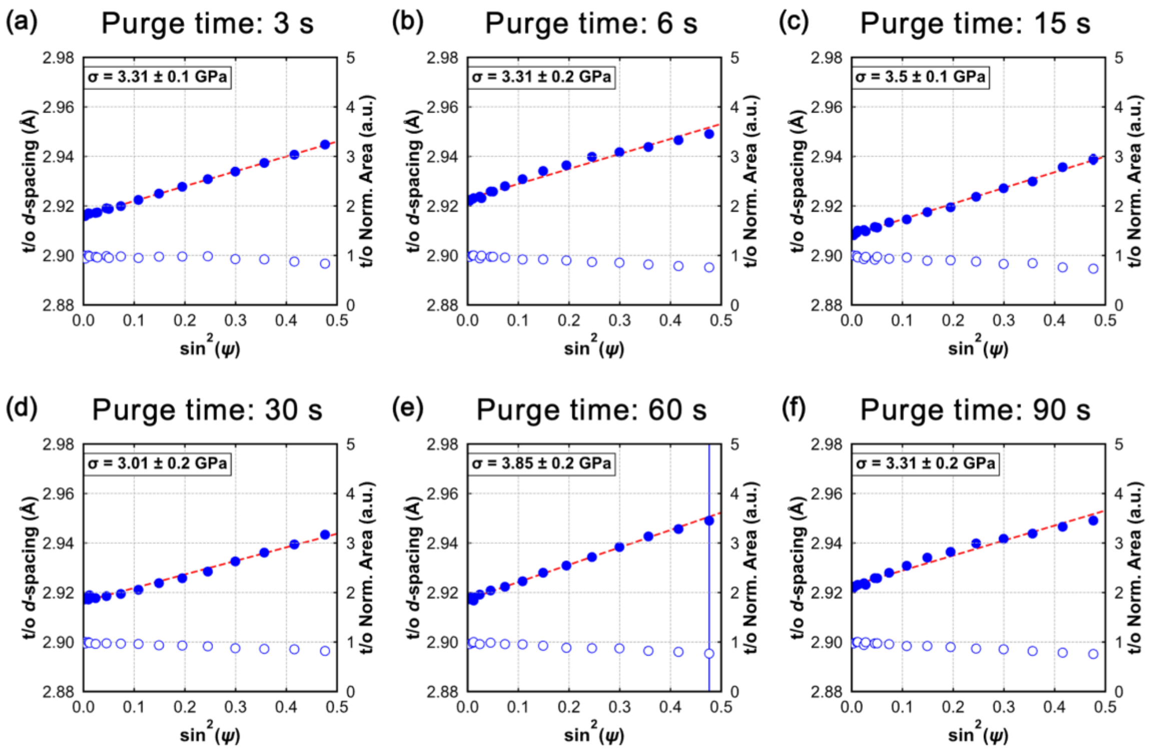

Figure S4. Metastable peak *d*-spacing (closed points, left axis) and normalized intensity (open points, right axis) and the associated linear fit (red lines) utilized to calculate the post-processing biaxial stress states for the metal precursor purge time with (a) 3 s, (b) 6 s, (c) 15 s, (d) 30 s, (e) 60 s, and (f) 90 s.

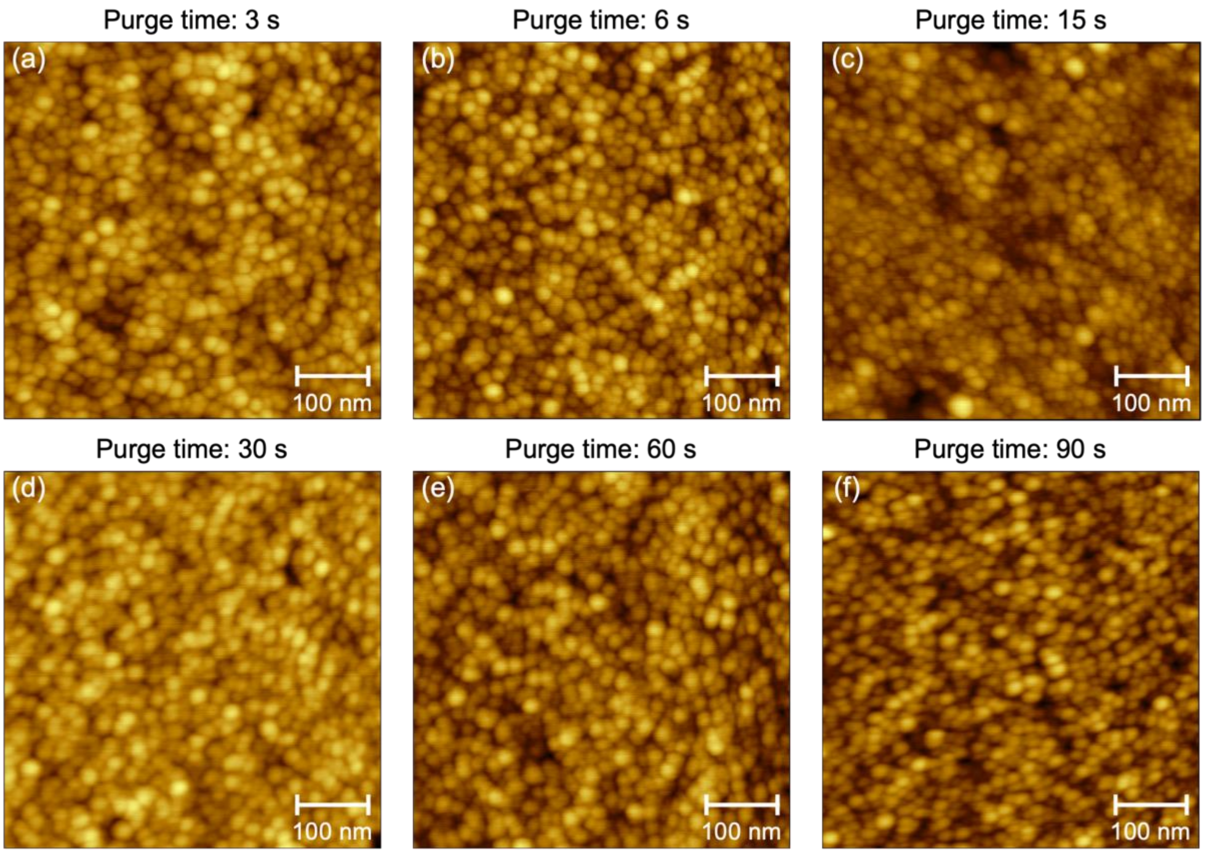

Figure S5. AFM topography images of HZO devices with different metal precursor purge time with (a) 3 s, (b) 6 s, (c) 15 s, (d) 30 s, (e) 60 s, and (f) 90 s.

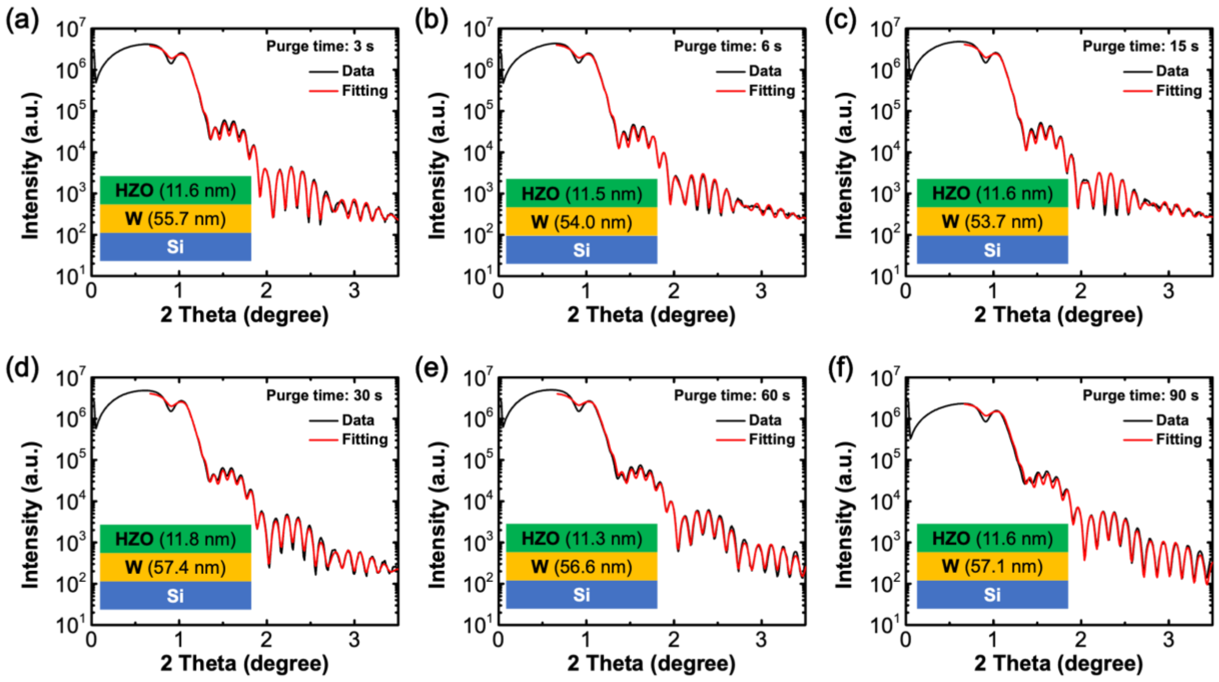

Figure S6. XRR patterns (black line) and associated fits (red line) corresponding HZO devices with different metal precursor purge time with (a) 3 s, (b) 6 s, (c) 15 s, (d) 30 s, (e) 60 s, and (f) 90 s. The inset image shows a schematic of the layers with thicknesses derived from XRR fitting.

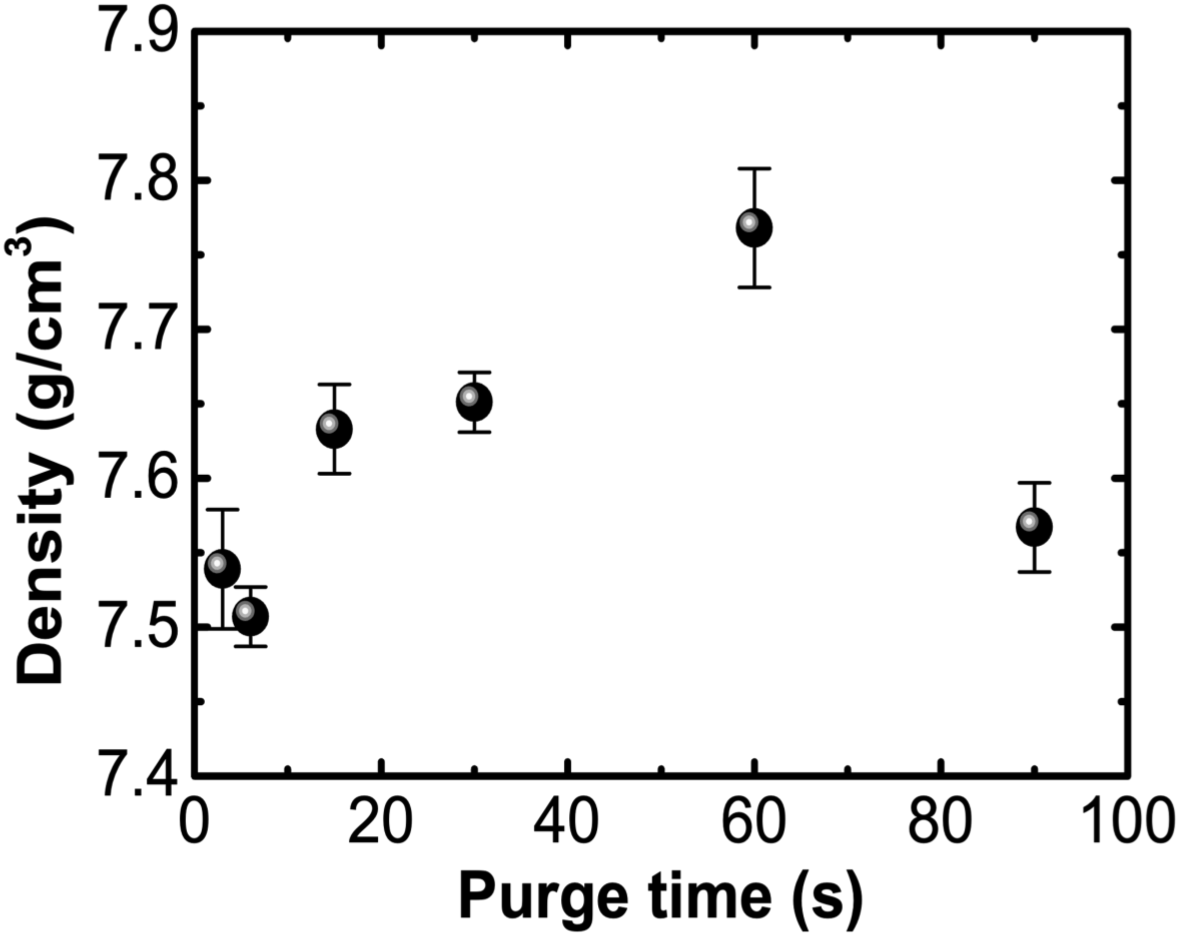

Figure S7. HZO film density calculated from XRR fitting of HZO devices with each metal precursor purge time.

XRR provides the thicknesses and densities of the HZO thin films, with the XRR data and fits shown in Figure S6 and the extracted densities in Figure S7. According to Figure 8 (a), the Hf to Zr ratio decreases as the metal precursor purge time increases, and considering the density of each, the density of HZO also decreases as the purge time increases (theoretical densities of HfO_2 and ZrO_2 are 9.68 g/cm^3 and 5.68 g/cm^3, respectively), but the results of XRR do not show a linear trend. This is different from the typical result of decreasing the Hf to Zr ratio, which suggests that the antiferroelectricity produced by decreasing the metal precursor purge time is due to a change in the HZO crystal structure other than the effect of the Hf to Zr ratio.

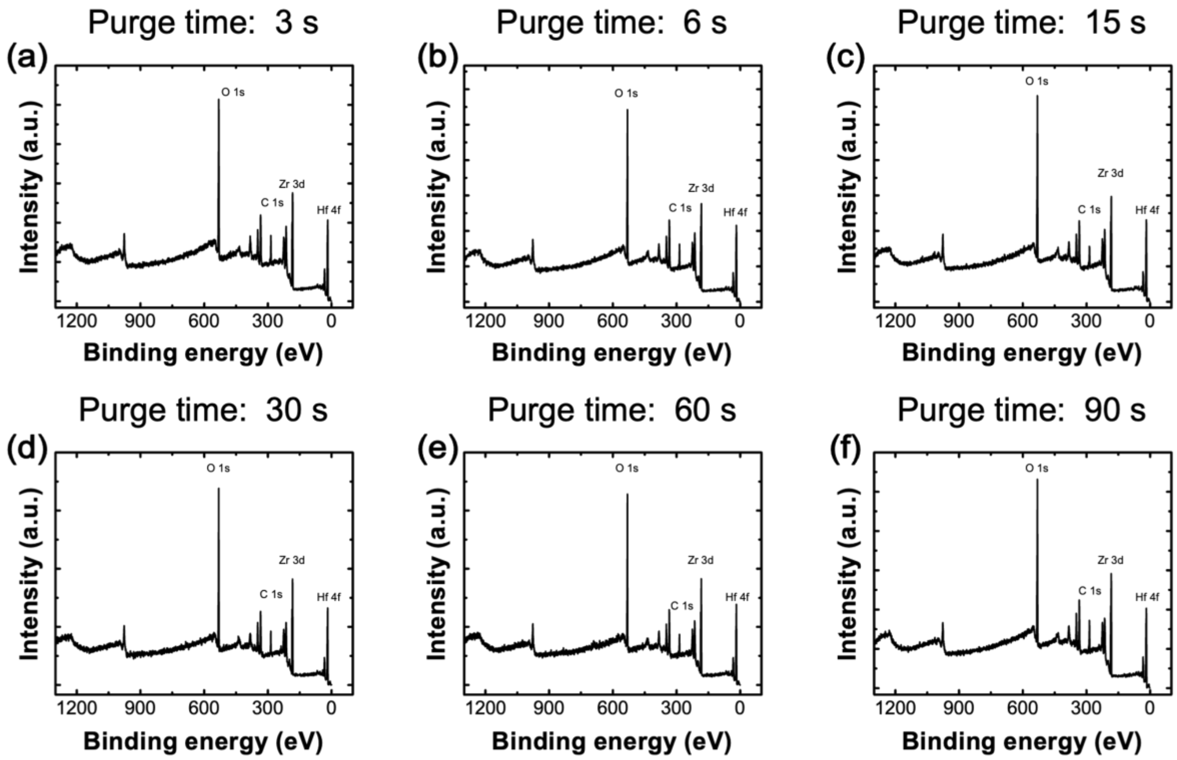

Figure S8. X-ray photoelectron spectra of survey spectra for HZO devices with precursor purge times with (a) 3 s, (b) 6 s, (c) 15 s, (d) 30 s, (e) 60 s, and (f) 90 s.

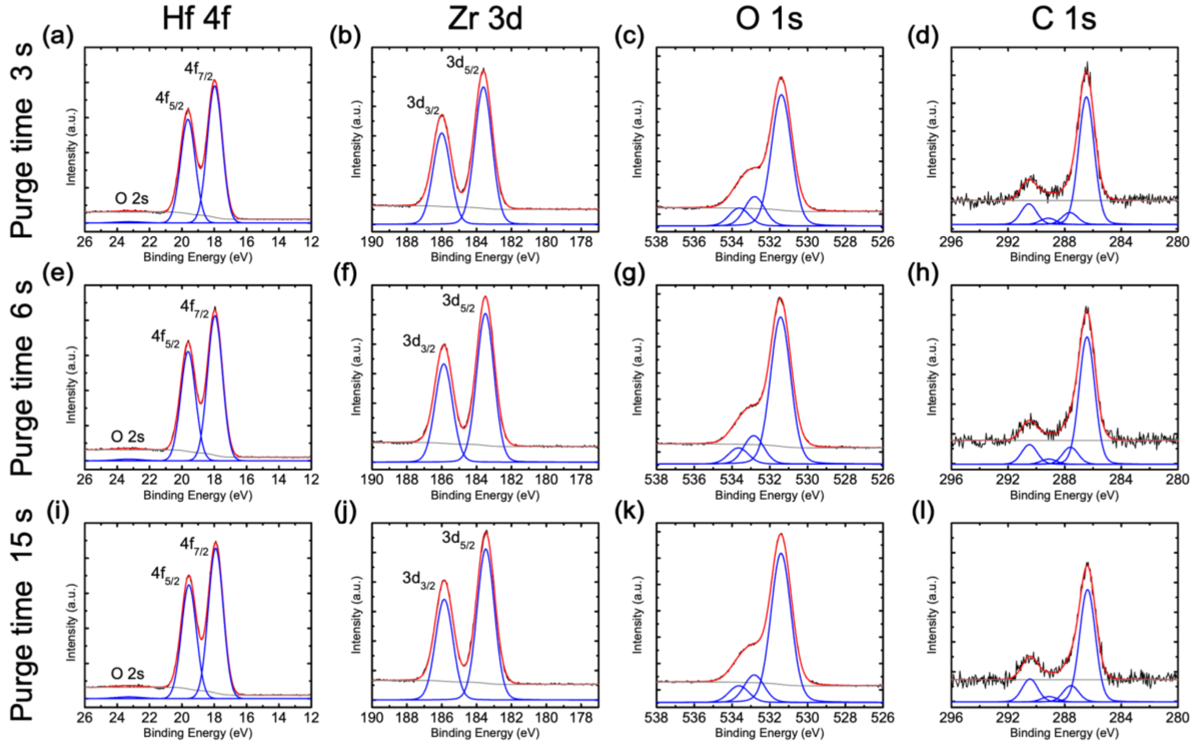

Figure S9. X-ray photoelectron spectra showing Hf 4f, Zr 3d, O 1s, and C 1s for HZO devices with precursor purge time with 3 s (a-d), 6 s (e-h), and 15 s (i-l). Raw signal is in black, total fit is in red, background fit in gray, and peak fits in blue.

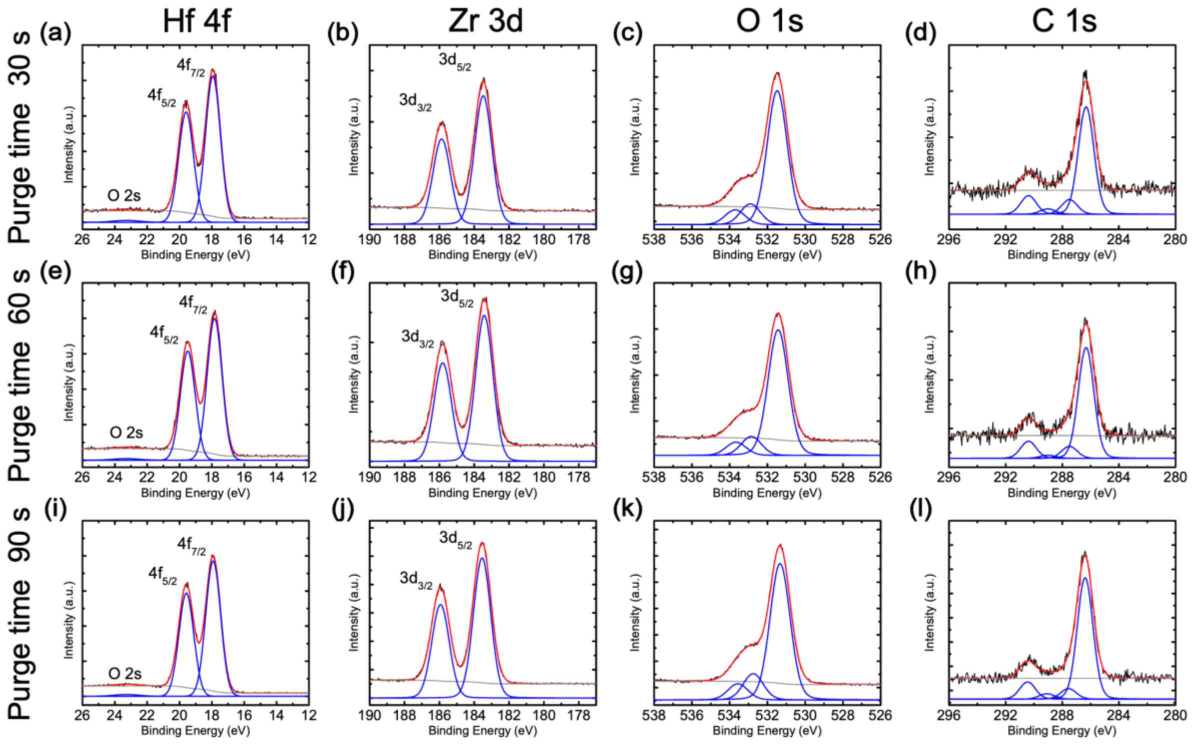

Figure S10. X-ray photoelectron spectra showing Hf 4f, Zr 3d, O 1s, and C 1s for HZO devices with different precursor purge time with 30 s (a-d), 60 s (e-h), and 90 s (i-l). Raw signal is in black, total fit is in red, background fit in gray, and peak fits in blue.

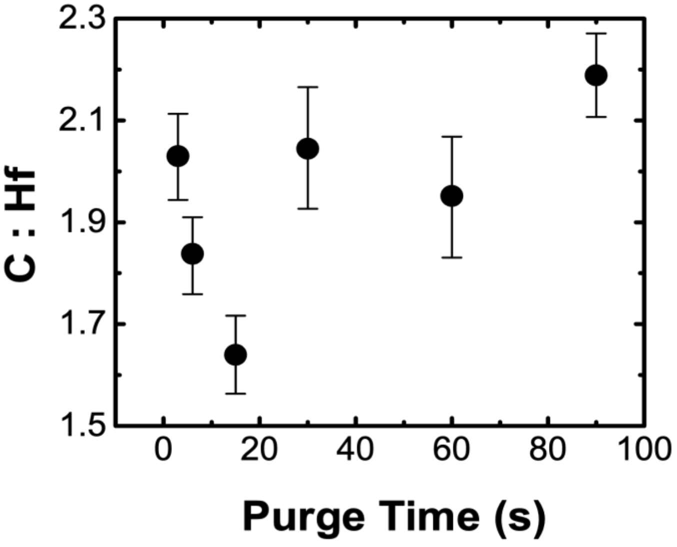

Figure S11. Relative total concentration of carbon to hafnium ratio with respect to metal precursor purge time from XPS.

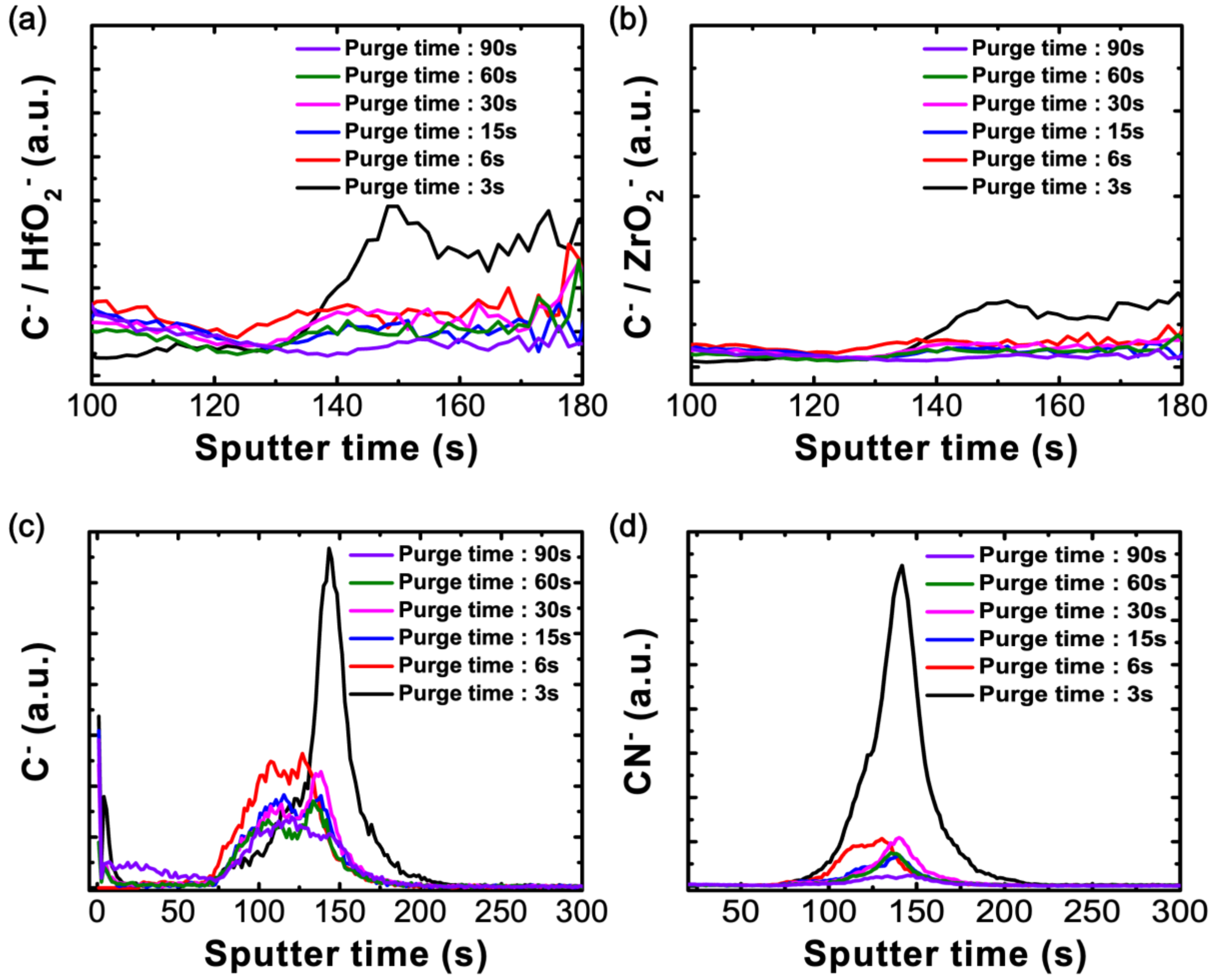

Figure S12. ToF-SIMS depth profiles showing (a) C^- divided by HfO_2^-, (b) C^- divided by ZrO_2^-, (c) C^- raw signal, and (d) CN^- raw signal with different metal precursor purge time
